# HUMANISE: Human-Inspired Smart Management, towards a Healthy and Safe Industrial Collaborative Robotics

**DOI:** 10.3390/s23031170

**Published:** 2023-01-19

**Authors:** Karmele Lopez-de-Ipina, Jon Iradi, Elsa Fernandez, Pilar M. Calvo, Damien Salle, Anujan Poologaindran, Ivan Villaverde, Paul Daelman, Emilio Sanchez, Catalina Requejo, John Suckling

**Affiliations:** 1 Department of Psychiatry, University of Cambridge, Cambridge CB2 3PT, UK; 2 EleKin Lab, Systems Engineering and Automation, Computers’ Architecture and Technology, and Enterprise Management Departments, University of the Basque Country UPV/EHU, 20018 Donostia-San Sebastian, Spain; 3 Tecnalia Research Centre, Tecnalia Industry and Transport Division, 20009 Donostia-San Sebastia, Spain; 4 The Alan Turing Institute, British Library, London NW1 2DB, UK; 5 Department of Mechanical Engineering and Materials, Engineering School, University of Navarra, TECNUN, 20018 Donostia-San Sebastian, Spain; 6 CEIT, Manufacturing Division, 20018 Donostia-San Sebastian, Spain; 7 Cajal Institute, Consejo Superior de Investigaciones Científicas (CSIC), 28002 Madrid, Spain

**Keywords:** Cobot, Machine Learning, risk management, human/robot behaviour, ageing population, workers’ diseases, industrial health and safety

## Abstract

The workplace is evolving towards scenarios where humans are acquiring a more active and dynamic role alongside increasingly intelligent machines. Moreover, the active population is ageing and consequently emerging risks could appear due to health disorders of workers, which requires intelligent intervention both for production management and workers’ support. In this sense, the innovative and smart systems oriented towards monitoring and regulating workers’ well-being will become essential. This work presents HUMANISE, a novel proposal of an intelligent system for risk management, oriented to workers suffering from disease conditions. The developed support system is based on Computer Vision, Machine Learning and Intelligent Agents. Results: The system was applied to a two-arm Cobot scenario during a Learning from Demonstration task for collaborative parts transportation, where risk management is critical. In this environment with a worker suffering from a mental disorder, safety is successfully controlled by means of human/robot coordination, and risk levels are managed through the integration of human/robot behaviour models and worker’s models based on the workplace model of the World Health Organization. The results show a promising real-time support tool to coordinate and monitoring these scenarios by integrating workers’ health information towards a successful risk management strategy for safe industrial Cobot environments.

## 1. Introduction

Humanity is facing a new labour environment where the highly-qualified active population is ageing, and highly-qualified young population have unstable employment. Moreover, working environments are becoming increasingly stressful, requiring higher cognitive demands from individuals due to industrial and technological workplace pressures. Additionally, while traditional psychosocial risks may arise from a variety of novel working conditions, the traditional job content/context model does not apply to the modern and ‘smarter’ workplace. Therefore, preventive workplace measures sometimes may not be applicable to these new working scenarios. Furthermore, new emerging factors should be carefully integrated into workplaces because work is a valuable element for healthy active ageing. In this regard, cognitive well-being is crucial for an efficient and satisfactory working life [1].

Ageing induces health impairments in the three aspects of health defined by the World Health Organization (WHO) [2]: physical, mental and social. Additionally, cognitive impairments start in later life, and in the early stages it can lead to diseases such as anxiety, depression, and in more advanced stages Alzheimer’s and Parkinson’s that produce a clear negative impact on workers’ performance. Conversely, increasing social difficulties such as caring for dependants, work-life imbalance, exclusion risks and immigration, increase the complexity of current working environments.

**Figure 1:**
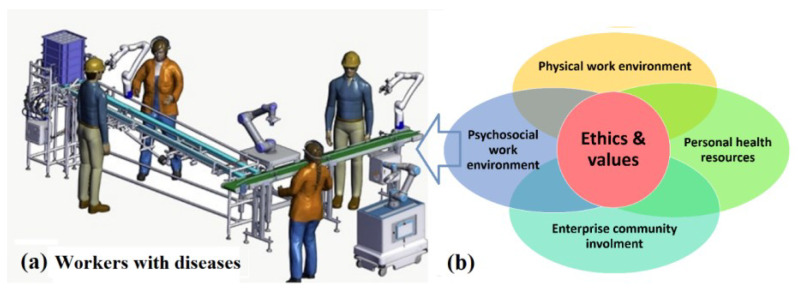
HUMANISE’s framework consists in the management of safety in Industrial Collaborative Robotics with workers suffering from disease conditions: (**a**) Industrial Collaborative Robotics (CoBot) scenario. (**b**) Worker’s health conditions through World Health Organization (WHO) workplace model [2].

In these complex workplaces, innovative tools are capable of promoting cognitive well-being, which is crucial to maintaining healthy ageing and to contributing to the economy and society as highlighted in the reports of the World Health Organization that provides an essential tool to describe environments in terms of healthy workplace models (Figure 1). Within these scenarios, several labour risks are identified, among others: (a) increased fatigue due to the loss of cognitive well-being because of multiple work and psychosocial factors, (b) early cognitive impairment (early neurodegeneration that is becoming epidemic MCI (Mild Cognitive Impairment), PD (Parkinson’s Disease), (c) increased levels of stress (anxiety), and (d) increased fragility and chronicity of the worker’s health. Subsequently, the following issues need to be tackled, which results in a great increase in occupational risks and leaves for medical reasons: (a) deterioration of mental health (anxiety and depression disorders), (b) fragility, (c) chronicity, (d) lower performance, and (e) loss of functional capacity, performance and production as well as lower quality output [1,2,3].

These conditions have a serious negative impact on family and working life: precariousness; reduced access to health support; more competitive and isolated working environments; increase in psychosocial stress levels (tele-stress); and increase in emerging risks. Taking into consideration the negative impacts, some workplaces have evolved to incorporate home-based teleworking that helps to balance family-work life, which usually affects women with care responsibilities [4]. The COVID19 current situation has accelerated all these changes. Therefore, as stated by the European Agency for Safety and Health at Work remark [5], Information and Communication Technologies (ICTs) and intelligent machines such as robots that provide automatic human skills will undoubtedly bring major new social and economic opportunities as well as emerging risks, helping to decrease the digital and work gaps (gender, salary, [1,4]).

It will be essential to make individuals aware of the importance of these changes to the workplace, and to increase the adaptation capacity of workers by means of innovative supporting tools that also guarantee personal privacy and data protection without increased technology cost [2,3,4]. This framework brings challenges into working environments and smart systems based on Artificial Intelligence and Collaborative Robots (Cobots) that will become essential resources for workplaces and industry.

Innovative and safe workplaces involving Cobots will be oriented towards monitoring and regulating the workers’ activity, safety and security, while also trying to promote a more proactive and healthier lifestyle. The new cognitive platforms for smart Cobot operation will include: smart management, regulation and support, safety and security control, quality and productivity improvement, workers’ support and/or intelligent learning scenarios. These novel machines require complex and intelligent human skills such as precision, planning, motion, complex perception, flexibility, versatility and intelligent control. In fact, current systems try to imitate human behaviours by integrating vision, prediction or intelligent management, and in the near-future will also likely impersonate complex brain processes [5].

In recent years, several industries have brought robotic technology into their markets: household robots, entertainment robots, logistics robots, public environment robots, defence applications, inspection and maintenance robots, professional cleaning robots, agricultural robots, motorized human exoskeletons, construction and demolition robots [6]. In the flourishing field of medical robotics, significant advancements include: robotic surgery which promotes the transition from open to laparoscopic surgery and other minimally invasive surgical procedures [7], bionic prostheses, and caretaker robots [8].

The opportunities for robotics and autonomous systems in the agricultural-food production include: the development of field robots that can assist workers by carrying payloads and conduct agricultural operations such as crop and animal sensing, weeding and drilling, or integration of autonomous systems technologies into existing farm operational equipment such as tractors [9]. These examples together demonstrate that the boundaries between industrial and service robotics are blurring [10], and that human-robot interactions are assuming a broader role, and becoming applicable to a wider variety of applications where a closer interaction between humans and robots is anticipated.

Human-Robot Collaboration (HRC), is favoured by two cognitive abilities: intentional reading and confidence. A robot possessing these abilities could infer the non-verbal intentions of others and assess the likelihood that they will achieve their goals, jointly understanding what type and degree of collaboration they require [11].

This work is part of a research line focus on the improvement of efficiency and safety in industrial Cobot environments [12,13,14] and presents the first approach and preliminary results of HUMANISE, a human inspired smart management system for healthy and safe industrial collaborative robotics (Cobots). The system integrates the WHO workplace model with principles from Machine Learning (ML), intelligent agents, and fractional control (Figure 1). The paper is organized as follows: Section 1 contains the introduction; Section 2 describes Industrial Cobots; Section 3 includes methods and materials; Section 4 presents the results and discussion. Finally, the concluding remarks are presented in Section 5. Additionally, this work will be framed against the backdrop of an Ethical, Risk Management, and Regulatory framework for the long-term implications of Cobots technology, and thus will include user-oriented and universal design methodologies.

## 2. Collaborative Robot (Cobot) in Industrial Environments

**Figure 2:**
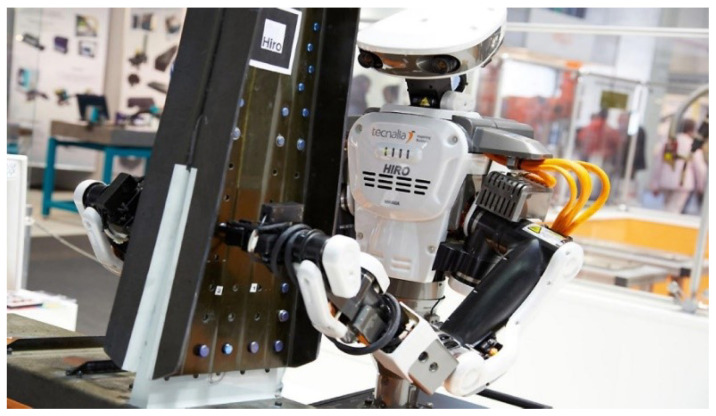
Robot Kawada Nextage Open of Tecnalia [13].

Cobots are robots designed to automate production processes by sharing a work environment through human-machine interaction (Figure 2). Due to the inherent risks of production processes, safety is a critical factor when designing both the collaborative work mode and the cooperative workspace [12,13,14,15,16,17]. This integration is occurring in several automated elements: Cobots, intelligent systems, high precision automation, augmented reality, bionic (wearable exoskeleton or prosthesis) or health robotic systems (neurosurgery, neurorehabilitation).

The notion of HRC, based on the correlative work of robots and humans, raises questions about the traditional model of physical barriers that separate machines and workers, and highlights the dwindling gap between both behaviours. There are two technological factors that have made this paradigm shift possible: (1) the integration of safety-related features in the architectures of robots and control systems, and (2) the use of multimodal interfaces for a more intuitive, conscious, and safe Human-Robot Interaction (HRI) [18,19]. Cobot systems are a new robotics technology that allows robots and human operators to work together in ways that were previously impossible. There are four different types of collaborative operations defined in the ANSI/RIA robot safety standard documents [20,21] (1) Safety-rated Monitored Stop (SMS); (2) Hand Guiding (HG); (3) Speed and Separation Monitoring (SSM), and (4) Power and Force Limiting (PFL).

Therefore, it is very important that Cobots include safety standards that reduce risks. Although Cobots have built-in security measures that allow safe applications, this state often changes as soon as they are integrated into a work environment. Therefore, safety systems must be installed to avoid risks for humans that the robot may generate, as well as safety measures related to the design of the work cell [21]. Moreover, future Cobot systems will not only need to adapt to workplace conditions, but also need to incorporate features of the worker’s mood, health, and associated factors in order to design robust control systems of the workplace, and ultimately mitigate risks [22,23,24,25].

According to some studies, correctly predicting human actions through the understanding of human intention can produce safer human-robot interactions [25] and more efficient human-robot teams [26]. Furthermore, it was found that robot planning using a human planning model can produce plans that can be generalized better than plans learned without such a model [27]. Other works have developed methods to predict human intention based on low-level human movement [28,29,30] as well as higher-level models of human reasoning based on modelling of social forces [31]. If we look at the safety assessment in the HRC, some approaches use objective measures to plan and evaluate the performance of applications that present collaborative “speed and separation monitoring” scenarios [32,33]. However, they also demonstrate the difficulties in identifying the moment in time during a robot’s trajectory where a specific algorithm is the least secure, requiring simulation or testing with the entire system.

**Figure 3:**
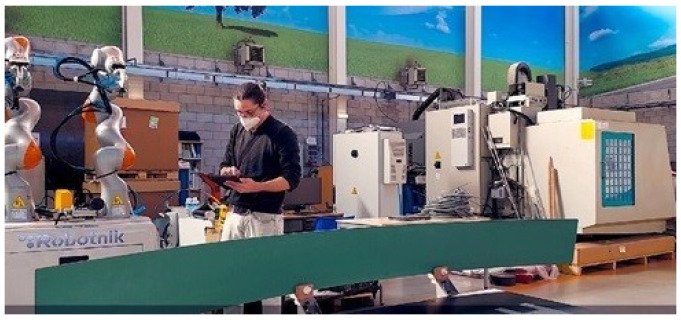
Scenario of the Use case 1: Cobot Learning from Demonstration (LfD).

In this sense, the LBR iiwa is the world’s first series-produced sensitive (HRC-compatible) robot. LBR stands for Leichtbauroboter (German for lightweight robot) and iiwa for intelligent industrial work assistant [34]. LBR iiwa provides the necessary skills for humans and robots to work in close cooperation with a high effectiveness and efficiency. The Kuka LBR iiwa robots with a payload of 7 kg are equipped with torque sensors in each joint. These robotic arms can provide force and torque information estimated from the torque sensors placed in each joint. In [12] authors worked on identifying dynamic parameters that can predict the robot joint torques by using global optimization. Furthermore, the previous works with this Cobot were focused on new schemes for safety and real-time feedback of the process state in a framework of cooperative applications for industrial environments (Figure 3). Thus, several architectures were developed to manage flexibility, reusability with multisensory intelligent robots [13] and the execution of trajectory driven collaborative tasks by combining control, trajectory coordination and effective robot-to-human feedback [14], and by a path-driven mobile co-manipulation architecture definition of safe and collaborative areas [15]. Moreover, these tasks are also in SHERLOCK project [16], which oriented the latest safe robotic technologies including high payload collaborative arms, exoskeletons and mobile manipulators in diverse production environments.

## 3. Materials and Methods

### 3.1. Materials

The materials consist of a high-resolution video (Around 5000 frames, 24 frames/s, image size 1080 × 1920 pixels) of a real task recorded in Tecnalia’s flexible robotics laboratory. Specifically, a demonstration cell was developed, which was designed to teach the possibilities offered by Cobots. The deployed Cobots were Kuka LBR iiwa (two arms) on a mobile platform with and external IO module.

In this work, in the framework of risk management in Cobot environments for ageing and workers suffering from diseases, the so-called “collaborative parts transportation” was selected because many industrial sectors require moving large parts among different areas of the workplace, using a large amount of the workforce. Furthermore, one of the most relevant HRC tasks was selected, the “Cobot Learning from Demonstration (LfD)” task. LfD refers to the process used to transfer new skills to a machine by relying on demonstrations from a user. This task is inspired by the imitation process of humans and animals to acquire new skills. LfD provides an easy and intuitive way to give new skills to the Cobot [35]. Specifically in this work during the LfD task, the worker demonstrated to the Cobots how to manage and transport large parts. The worker showed to both Cobots how: (i) to pick the large part (positions); (ii) to move it through a pathway to the next workstation; (iii) deposit it; (iv) the Cobots should coordinate in each step of the task. This is the Use case 1 and it was a coordinated Cobot learning task between the two-arms of Cobot and a worker (Figure 3). The monitoring video sequences acquired by the external high-quality camera were processed by a custom toolbox in MATLAB to create activity time-series [36,37].

Three Use Cases (UC) oriented to risks generated by worker’s stress were created and evaluated, the recorded real-task and two simulated tasks:

1. UC1-Coordination: this is the previously described real task, Cobot LfD (Figure 3) for “collaborative parts transportation”.
2. UC2-Stress: this was a simulated case of a stressed worker based on the visual feedback features of UC1. An algorithm generated this simulated case.
3. UC3-High risk: this was a Cobot risk behaviour UC where the Cobots did not perform in a coordinated way, and there was high speed and accelerated misbehaviour. An algorithm generated this simulated case.

Summing up, this work presents the preliminary results of the HUMANISE system evaluated over these three UCs and information of a Mental Disorder (anxiety). Moreover, the three UCs were evaluated over two LfD tasks with different risk levels: (i) Extra Large size parts with low speed (LfD-XL); (ii) Large size parts with medium speed (LfD-L).

### 3.2. Methods

HUMANISE is oriented to current and future industrial scenarios where flexibility and versatility for small series, co-feedback and open collaboration among Cobots and humans, human-robot interaction, real-time applications and/or safety are essential requirements.

In these working environments, emerging risks appear, and intelligent support is required for both management and control of machines and production. In this work, we develop HUMANISE, an intelligent management system of human operation and robotic behaviour for healthy and safe industrial use. The developed systems and tools are based on computer vision and artificial intelligence and are applied to a two-arm Cobot scenario during a learning process for flexible and versatile manufacturing.

#### 3.2.1. Overall Methodology and Framework

**Figure 4:**
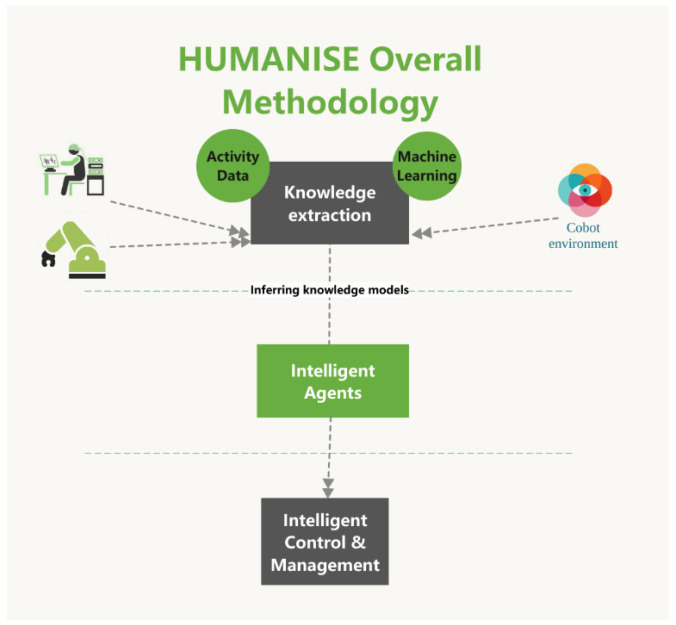
Diagram of the overall methodology of HUMANISE.

The framework of HUMANISE is oriented to the management of safety in industrial collaborative robotics with workers suffering from disease conditions. Thus, the main objectives were focused on the following measurable objectives by integrated phases (Figure 4):

1. Knowledge extraction: (i) Knowledge from the WHO health workplace models, (ii) Knowledge from the environmental activity data of the visual feedback provided by the perception system, (iii) Knowledge provided by the ML models.
2. An intelligent agent to provide smart interactive support to a specialist who could interact and regulate the environment. The agent will be guided in a future design by means of human-brain patterns.
3. Intelligent control and management of the Cobots by the regulation of both control algorithm parameters and trajectory coordination.

#### 3.2.2. Smart Management System

**Figure 5:**
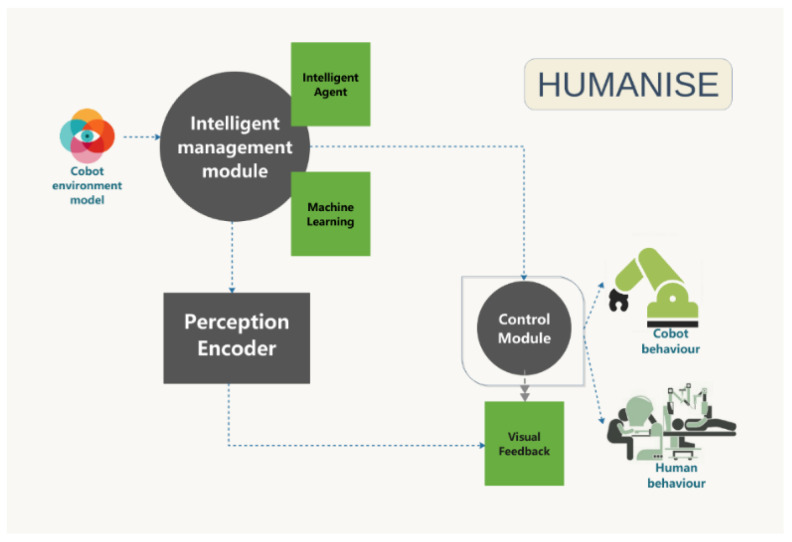
Diagram of the Smart Management System.

The overall objective of HUMANISE is to develop a smart system to manage Cobot working environments whilst supporting workers to improve their health and safety conditions (Figure 5). Specifically, the project will establish a working synergy between several broad and multidisciplinary areas: health and prevention of occupational risks, neurology, psychiatry, active ageing, health care, telecommunications, modelling and control, and computer science. These ML and intelligent methodologies will predict and analyse workplace situations. Figure 4 shows the block diagram of the Smart Management System of HUMANISE. The architecture of the system is based on a closed loop system with visual feedback and intelligent management of the performance conditions.

The main components of the system are:

1. The intelligent management module: the core of this module consists of the WHO and visual environment ML models (Support Vector Machines, SVM), and an intelligent agent. This last component will manage the conditions of the workplace by sending instructions and information to the control module.
2. The control module: This component adapts the Cobot behaviour and gives support to the workers for a successful and coordinated evolution of the task improving the quality of the process. This real-time control and support will be essential for health and safety work conditions.
3. The perception encoder. This component manages in real-time the visual information that enriches the intelligent management module.

#### 3.2.3. WHO Health Workplace Model

HUMANISE also regulates conditions by a smart management system, which integrates ML modelling based on WHO’s health workplace model (Figure 1) that includes:

1. Health and safety in the physical work environment.
2. Health, safety, and well-being in the psychosocial work environment.
3. Personal health resources in the workplace.
4. Enterprise community resources.

#### 3.2.4. Monitoring and Control of the Cobot Environment

**Figure 6:**
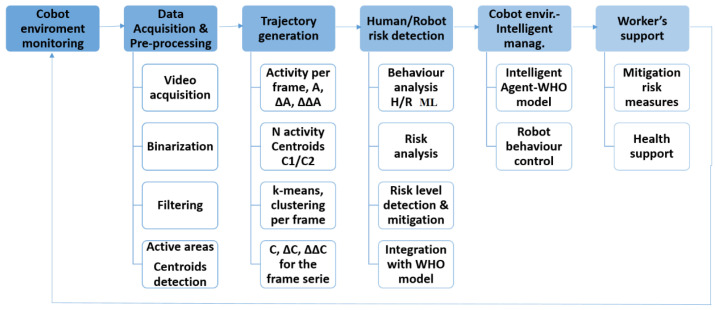
Workflow of the Cobot environment: monitoring and control, showing the steps of image acquisition, pre-processing, data treatment, Human/Robot (HR) behaviour analysis and intelligent management of the environment through WHO models to support the workers.

Figure 6 shows the Workflow of the Cobot environment, which covers two main tasks:

1. Monitoring: The monitoring of the activity areas is used as co-manipulation feedback and support for workers in risk.
2. Intelligent risk management: Robot/Human (R/H) behaviour management, robot control and worker support.

The main activities in the process are:

- Cobot environment monitoring: the main information is provided as feedback.
- Data/image acquisition and pre-processing: acquisition, binarization and filtering for each frame, detection of activity areas and extraction of activity centroids for each area.
- Trajectory generation: (i) for monitoring, extraction of n regions of interest (ROIs) of the activity. (ii) for risk management, trajectory generation and trajectory feature extraction.
- Human/Robot (HR) risk detection: HR behaviour analysis and integration with the WHO models.
- Cobot environment intelligent management: intelligent management of the environment by the agent with information of the WHO models. Robot control by the information of coordination.
- Worker support: the system will provide support and also feedback by the monitoring system.

In the next sections the main activities are described in detail.

##### Data Acquisition and Pre-Processing

The monitoring video sequences were acquired by a high-quality camera, and processed by a custom toolbox in MATLAB in order to create activity time-series [36,37,38]. Then, during the pre-processing the activity areas (motion detection) were calculated based on a frame difference method (Frame absolute difference) between two consecutive frames. Activity detection and motion estimation were performed in order to detect HR moving and simultaneously eliminate background, noise and artifacts.

In the next step, images were divided into n regions of interest (ROI) in order to monitor each with the mean activity of the pixels within the ROI for real time processing, and converted to binary images, and then the main activity areas were defined applying morphological filters to reduce the noise and for smoothing. Monitoring of the Cobot environment activity: (a) Image of activity difference in two consecutive frames. (b) Activity (A) in a frame. (c) ∆A in a frame (d) ∆∆A in a frame.

##### Feature Extraction: Trajectory Analysis

The evolution and coordination of the components in the system (Cobots and human) was modelled by the trajectories of N activity centroids along time. In this sense as in [36,37,38] k-means algorithm was selected because the centroids need to capture the information provided by the main activity areas in order to analyse the environment coordination. In addition, as in previous works [36,37,38,39,40] there was essential knowledge on the task (R/H activity distributed by areas, behaviour features,) and also there were critical requirements of real-time. Thus, within each frame in the binarized image, the coordinates of the centres of all the activity areas were calculated and then k-means was applied to find the centroids of the activity clusters (centres, with x and y coordinates).

Finally, the activity was analysed in real-time by the following features for each centroid and each coordinate (x and y):

- Number of activity areas.
- Velocity, and speed (∆).
- Acceleration (∆∆).

A dataset was created with the features of the trajectories of the centroids generated for all the frames (time-series).

##### HR Behaviour Modelling and Risk Management

We will develop a hypothesis-based modelling. In this sense, the modelling is based on the evolution and coordination of the system that is represented by the evolution and coordination of N centroids. Specifically, In the UCs for this controlled scenario, there could be two centroids mainly focused on the worker and the Cobot. Thus, ML classifier algorithms were selected taking into account the knowledge about the task and the robustness of these classifiers for real-time and noise environment requirements oriented to a binary case [41,42,43,44,45,46]. The scenario’s classes for modelling are the number of centroids (NC =2, C1 and C2).

Then in order to model the system in real-time conditions where the performance time is critical, two classifiers were used:

1. Support Vector Machines (SVM).
2. Multilayer Perceptron (MLP) defined by L layers of N neurons and the Number of Neurons in Hidden Layers (NNHL): L = 1 and NNHL= (number of features + classes number)/2.

Finally, the information provided by the ML models is integrated with the WHO models of the worker, and an intelligent agent manages the coordination and risk levels. In addition, this last component will manage the conditions of the workplace by sending instructions and information to the control module and worker support for feedback and coordination.

##### Intelligent Management of the Cobot Environment and Worker Support

Subsequently, these features were managed in the control module, this module adapts these features to control the robot behaviour. Then, integrating the previous features in the models, the intelligent agent sends the information to manage the fractional and classical Proportional-Integral-Derivative (PID) control algorithms that were used in the control module to adapt the Cobot behavior and to support the worker in order to adapt the work for a successful evolution of the task.

**Figure 7:**
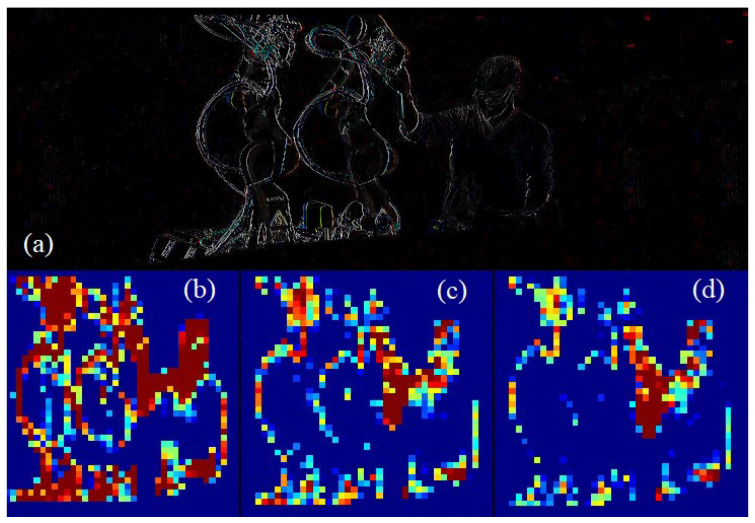
Monitoring of the Cobot environment activity: (**a**) Image of activity difference in two consecutive frames. (**b**) Activity (A) in a frame. (**c**) ∆A in a frame (**d**) ∆∆A in a frame.

Safety standards are included in the WHO model in the Enterprise community resources. The safety standards ISO 10218-1:2012 and ISO 10218-2:2012 describe the basic risks associated to robots and provide requirements to eliminate or adequately reduce the hazards associated with these risks. Cobots must also comply with ISO/TS 15066:2016 that specifies safety requirements for collaborative industrial robot systems and the work environment, and supplements the requirements and guidance on collaborative industrial robot operation given in ISO 10218-1 and ISO 10218-2 [47,48]. In addition, the worker can review the activity monitoring (Figure 7) and receives support and information to adapt her/his behaviour to improve the task performance.

#### 3.2.5. System Evaluation

We have used k-fold cross-validation with k = 10 for both training and validation of the ML models. Additionally, for the evaluation of the system the following criteria were used:

1. Coordination Level (CL, 0–100%). Accuracy (%) has been used in order to evaluate the CL between centroids. On the one hand, a value close to 50 in Accuracy is associated to a similar behaviour of both centroids, and this means a coordinated behaviour. On the other hand, as long as the value increases or decreases the CL decreases.
2. Risk Level (RL, 0–100%). RL = 100-CL. Then, when CL decreases RL increases and it needs to be regulated by the robot control system or by worker support.
3. Model Time (MT): This is the time to build the model and it is oriented to real-time systems. This is a critical parameter for the system in this framework.
4. ∆Risk Level: RL of the worker without HUMANISE management-Risk Level of the worker with HUMANISE management

#### 3.2.6. Calibration

Finally, the Kuka LBR iiwa calibration was also developed over the torque sensors in order to achieve an optimal performance. These robotic arms have the torque sensors placed in each joint in order to provide force and torque information. Calibration processes are essential when using sensors in robotic systems as they allow the data captured by the sensors to be related to the relative position of the robot in the work area. Calibration tools that are capable of improving the precision of a robotic manipulator, significantly reducing errors due to the real geometry of a robot by the use of a generic geometric model have been developed.

## 4. Results and Discussion

Figure 7 shows examples of monitoring: (a) an image of activity difference in two consecutive frames, (b) Activity (A) in a frame in n regions of interest (ROI), (c) the variation of A between two consecutive frames ∆A, and (d) the variation of ∆A, ∆∆A in a frame between two consecutive frames. Afterwards, this information was reduced by k-means to N centroids in order to manage the workplace conditions in real-time.

**Figure 8:**
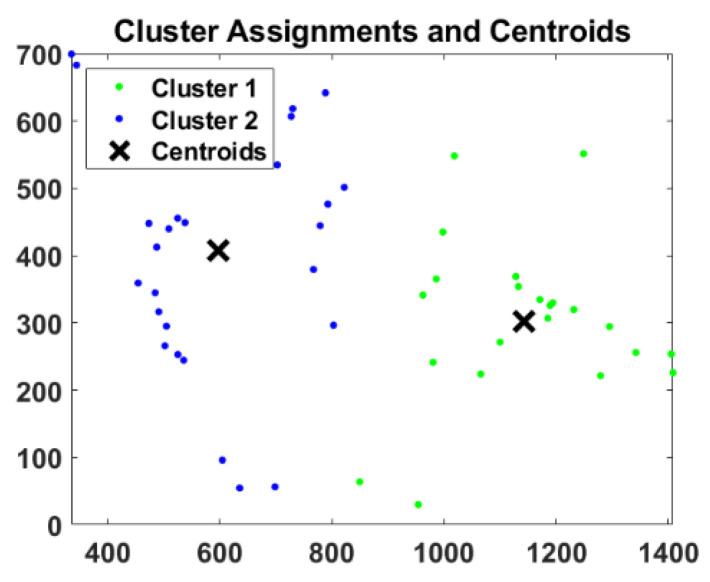
Generation of activity centroids by k-means, N = 2, Cluster 1 (C1) and Cluster 2 (C2) in a frame (frame size x in pixels, frame size y in pixels).

**Figure 9:**
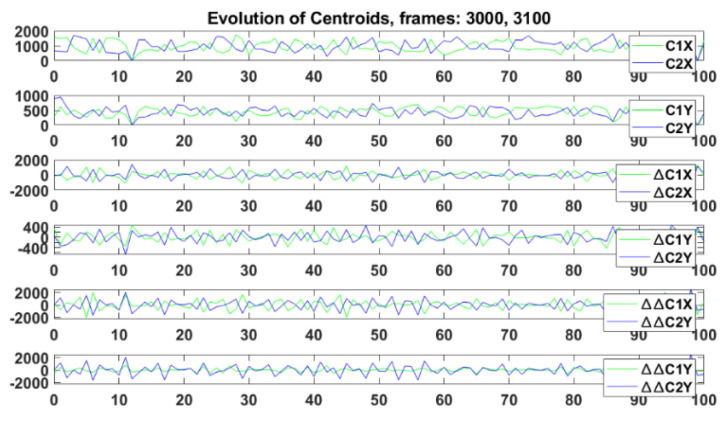
Analysis and evolutions of centroids during the coordinated task between the human and the Cobots, C1 and its coordinates and variation in green, and C2 and its coordinates and variation in blue. The variations along the time were calculated (difference between two consecutive frames) ∆C1 and ∆C2, and ∆∆C1 and ∆∆C2 were calculated for both coordinates (X, Y).

Specifically, in this first approach for the selected UCs, the centroid number in a frame was N = 2, Cluster 1 (C1), and Cluster 2 (C2), that models the human/Cobot behaviour. Figure 8 shows the results of the clustering. The clusters C1 and C2 covered the activity areas of the Cobot and the human worker. This process was repeated in each frame and produced time series of the evolution of the centroids that represent a real-time measurement of the evolution of the activity in the Cobot workplace. Afterwards, their variations along time were calculated (difference between two consecutive frames) ∆C1 and ∆C2, and ∆∆C1 and ∆∆C2 were calculated for both coordinates (X, Y). Figure 9 shows C1 and its coordinates and variation in green, and C2 and its coordinates and variation in blue. In this case, harmonious and coordinated behaviour was observed without large variations in the features. The greatest difference appeared for ∆∆.

**Figure 10:**
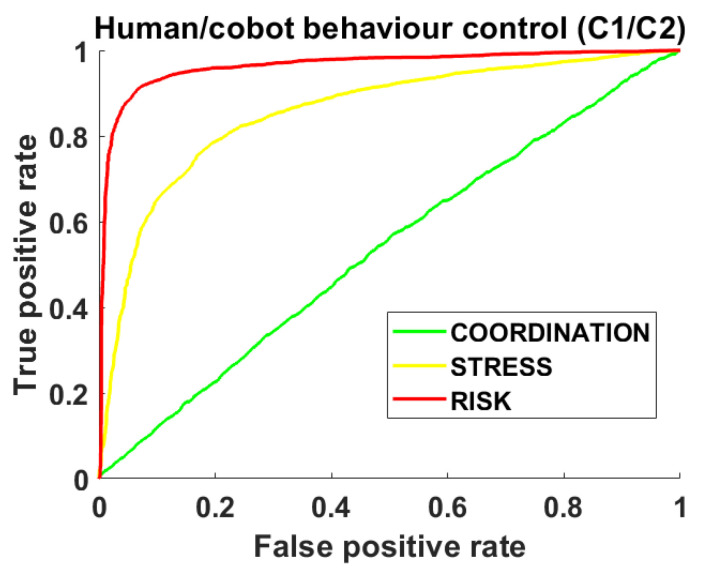
ROC analysis of the models of the centroids (C1, C2 by SVM) for the real and simulated use cases: (a) UC1: coordinated Cobot learning task in green. (b) UC2: Simulated stressed worker in yellow. (c) UC3: Risky behaviour of Cobots in red.

Figure 10 shows the results obtained for the three UCs by a ROC analysis of the SVM models of the centroids (C1, C2 by SVM) during the performance of the UCs (the real and simulated use cases): (a) UC1: coordinated Cobot learning task in green. (b) UC2: Simulated stressed worker in yellow. (c) UC3: Risky behaviour of Cobots in red. The ROCs showed clear differences for the UCs. Indeed, the values of Area Under the Curve (AUC) show the highest CL for AUC = 0.5 and an increase in this value for stress and risk conditions.

**Figure 11:**
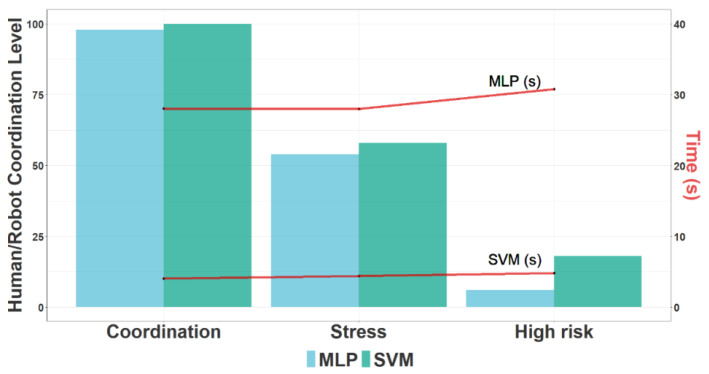
Results of Human/Robot Coordination Level for the three Uses Cases and two classifiers (MLP and SVM): UC1: Coordination, UC2: Stress, UC3: High risk. The lines in “red” indicate the Model Time for each classifier.

On the one hand, Figure 11 shows the results for the three Uses Cases and classifiers (MLP and SVM): UC1: Coordination, UC2: Stress, UC3: High risk. The lines in “red” indicate the Model Time (MT) for each classifier. The HR CL was properly detected in the three UCs with highest Accuracy for MLP. However, the differences for both classifiers were not significant whereas the Model Time was clearly higher for MLP. Thus, taking into account those factors and the real-time criteria, SVM was selected as the optimal model for this current framework.

**Figure 12:**
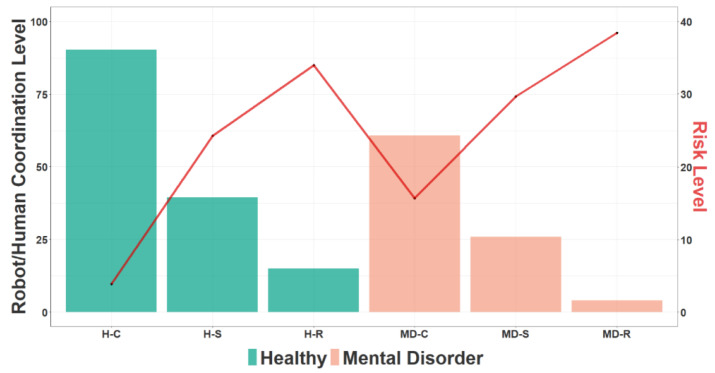
Results of the integration of the R/H behaviour models (SVM) and the WHO model for Mental Disorder (MD, anxiety) conditions for the three Uses Cases for (SVM): UC1: Coordination, UC2: Stress, UC3: High risk. The line in “red” indicates the Risk Level.

On the other hand, Figure 12, shows the results of the integration of the R/H behaviour models (SVM) and the WHO model for Mental Disorder (MD, anxiety) conditions for the three Uses Cases: UC1: Coordination, UC2: Stress, UC3: High risk. The line in “red” indicates the RL with an increase in the case of Mental Disorder when the CL decreases.

In the next step, the information was sent to the Smart Management Module that regulates the workplace information and the intelligent agent controls the parameters of the robot and sends support to the worker.

Finally, a comparison between the performance of workers using and without using the proposed system was carried out by simulation with health Real-World Data (RWD).

Remarkably, we could predict the RL along time (30 s), with the HUMANISE system (HUMANISE) and without (N-HUMANISE), over the two tasks LfD-XL and LfD-L under Stress (S) and Risk (R) conditions for a Healthy worker (H), a worker suffering from low-level anxiety (MD-L), and a worker suffering from medium-level anxiety MD-M. The RL was overall higher for LfD-L due to the higher speed of the Cobots and the increase in the coordination between the worker and the Cobots.

Under stress conditions, this preliminary study showed that HUMANISE decreases the RL of both tasks below the goal of RL = 50 before achieving a reference time of 30 s in most of the cases, with optimal results for H and MD-L. In this sense, MD-M achieved an optimal RL but slowly, with better performance for LfD-XL of lower speed and RL = 40.63 (15 s) in comparison with LfD-L with RL = 50.63 (15 s) over the threshold of 50.

Under risk conditions, only the healthy worker achieved an optimal RL in a short period. MD-L achieved an optimal RL but slowly, with better performance for LfD-XL of lower speed and RL = 32.03 (15 s) in comparison with LfD-L with RL = 42.03 (15 s). However, the MD-M worker is not able to manage the risk conditions, not even with HUMANISE support.

Thus, the higher complexity of the task, the worsening of workplace conditions or the anxiety level, produce a higher effort of HUMANISE that balances the RL towards optimum values which supports the safety and effectiveness of this model, making it a promising strategy to face additional risks considering multiple scenarios. Under risk conditions for the most complex task LfD-L, after 30 s without HUMANISE RL is 97.35 for the H worker and 100 for both the MD-L and the MD-M workers. In the workplace, under the same conditions using HUMANISE, the H and the MD-L workers can properly manage the task. However, HUMANISE cannot achieve optimum values in the case of medium anxiety levels where the reduction in RL is less than 40.

**Figure 13:**
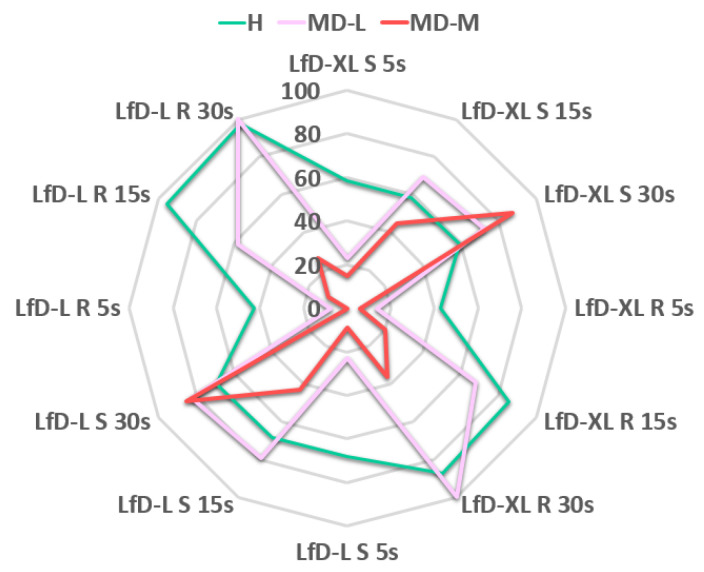
HUMANISE Risk Management (∆Risk Level Prediction) analysis along time (30 s) over LfD-XL and LfD-L for a Healthy worker (H, green), a worker suffering from low-level anxiety (MD-L, purple) and a worker suffering from medium-level anxiety (MD-M, red).

Figure 13 shows the improvement in Risk Management provided by the HUMANISE system. ∆Risk Level Prediction (∆RLP) represents the difference in the RLP with and without HUMANISE. ∆Risk Level Prediction along time (30 s) over LfD-XL and LfD-L for a Healthy worker (H, green), a worker suffering from low-level anxiety (MD-L, purple) and a worker suffering from medium-level anxiety (MD-M, red). The H worker had a more harmonic and adequate performance under all conditions and the MD-L worker had a faster adaptation and a more harmonic performance than the MD-M worker. The support provided by HUMANISE after 30 s improved RL conditions with higher ∆RLP after 30 s under complex conditions such as LfD-XL where the MD-M worker can properly manage the risk with the use of HUMANISE under stress conditions. However, under risk conditions for the MD-M worker HUMANISE is not able to decrease RL which remained above 50 with a low ∆RLP of 36.53 for LfD-XL and 26.53 for LfD-L.

These results indicate that a worker suffering from medium-level anxiety could require an adaptation of the workload and tasks, and personalized support mainly when the RL increases faster that the positive effect obtained by the support system due to the negative effects shown by the anxiety level.

These preliminary results show the promising performance of HUMANISE. The system provides a useful tool to manage a coordinated performance, not only of the two arms, but also of the worker integrated into the workplace. In line with these results, a recent study has proposed a novel methodology based on operating modes (“similar to a third-arm”) in order to implement a foot gesture command [49]. In addition to this, another recent study also showed an innovative, rapid and economical technology consisting of a 2-DOF robot arm with elastic behaviour which provides a safe human-robot interaction [50]. Remarkably, all these results suggest an improvement in manufacturing flexibility, and thus, support the advantages of developing Cobots for multiple application as long as they include a successful and economical operating system as in the case of KUKA LBR iiwa Cobots [51,52].

The application of novel methodologies to implement accurate systems to simulate the interaction between humans and robots advances the design of non-invasive strategies that provide medical support [53] or avoid risks in workplaces [54], such as collisions by using virtual sensors [55] or by human motion and behaviour prediction [56,57]. On the other hand, although Cobots are not yet fully accepted in industry, increasing evidence supports the valuable contribution of artificial intelligence in terms of feasibility and flexibility to achieve an improvement in security and communication [58,59]. Taken all together, the HUMANISE system goes one step further and enables acquisition and integration of information from different scenarios and behaviours to simulate real performance even under extreme situations.

The performance of the system could be improved in the future by increasing the intelligence of the agent by means of brain patterns to be able to decode, process and integrate environmental information to simulate and predict real human behaviours. In fact, the use of brain signatures has been proposed for analysing brain data previously collected by computational methods [60,61]. This novel approach could help with a better understanding of social human interactions, such as cultural transmission processes among others. In this context, non-verbal behaviour such as facial expressions, play an important role that must be taken into account. For this purpose, the combination of a standardized system, such as pre-trained convolutional neural networks (CNN) which extract information from facial expression recognition [62,63,64] along with our HUMANISE system, would be a promising tool for models that might give better diagnoses and improve the life quality of dependant people as well as improving working conditions. Finally, in the future brain patterns based on the analysis of fine motor skills will also be integrated in the WHO model [36].

## 5. Conclusions

Workplaces are clearly evolving towards future scenarios where humans acquire more active and dynamic roles integrating with increasingly complex machines that include among others: collaborative robotics (Cobots), intelligent systems, high precision control, augmented reality, bionic or social/health robotic systems (neurosurgery, neurorehabilitation). In these conditions, emerging risks appear, and not only intelligent control, but also intelligent support is required for both an active ageing population and workers suffering from diseases. This work presents the first version of HUMANISE, a novel system that integrates machine behaviour control and human support for healthy and safe environments. The system establishes a working synergy between multidisciplinary areas. Specifically, this work details the intelligent management of human and robot behaviour for safe collaborative robotics in an industrial Cobot environment of collaborative parts transportation. The developed systems and tools are based on ML, intelligent agents and computer vision, and were applied to a two-arm Cobot scenario during a LfD process for flexible and versatile manufacturing. The results are promising and provide a valuable, easy to use, and robust support tool for the management of Cobot workplace environments. The intelligent performance of such systems could be improved in the future by increasing the intelligence of the agent by deploying neuroscience-inspired algorithms.

## 6. Ethics Statement

The industrial standards for Cobot development have been applied. This work is framed by a discussion on ethical, risk management, and regulatory aspects for long-term implications of this technology, and thus user-oriented and universal design methodologies have been included [65].

## Data Availability

The datasets generated by and/or analysed during the current study are not publicly available due to ethics and privacy requirements, but they are available from the corresponding author upon reasonable request.
